# Oncogenic role of copper‑induced cell death‑associated protein DLD in human cancer: A pan‑cancer analysis and experimental verification

**DOI:** 10.3892/ol.2023.13800

**Published:** 2023-04-06

**Authors:** Han Qi, Dongsheng Zhu

**Affiliations:** 1 Department of Emergency Surgery, The Second People's Hospital of Lianyungang, Lianyungang, Jiangsu 222000, P.R. China; 2 Department of Paediatric Surgery, The First People's Hospital of Lianyungang, Lianyungang, Jiangsu 222000, P.R. China

**Keywords:** cuproptosis, dihydrolipoamide dehydrogenase, cancer, prognosis, pan-cancer analysis

## Abstract

Copper ions can bind directly to lipoylated components of the tricarboxylic acid (TCA) cycle, triggering the aggregation of mitochondrial lipoylated proteins and the destabilization of Fe-S cluster proteins, resulting in copper-dependent cell death. Dihydrolipoamide dehydrogenase (DLD) is a key protein of the TCA cycle and constitutes the E3 component of the α-ketoglutarate dehydrogenase complex, which is deeply interconnected with the mitochondrial electron transfer chain in the TCA cycle. Tumor cells demonstrate dependency on glutaminolysis fuelling to carry out the TCA cycle and essential biosynthetic processes supporting tumor growth. Therefore, DLD plays an important role in the tumor biological process. However, to the best of our knowledge, no pan-cancer analysis is currently available for DLD. Therefore, the present study first explored the DLD expression profile in 33 tumors in publicly available datasets, including TIMER2, GEPIA2, UALCAN, cBioPortal and STRING. TIMER2, GEPIA2 and UALCAN were used for exploring gene expression; survival prognosis was detected by GEPIA2; genetic alteration was analysed by cBioPortal; immune infiltration data was obtained from TIMER2; interacting proteins of DLD were detected by STRING. DLD was found to be highly expressed in colon, liver, lung, stomach, renal, corpus uteri endometrial and ovarian cancers compared with normal tissues, and its high expression was associated with poorer prognosis in ovarian cancer. To the best of our knowledge, the present study provided the first comprehensive pan-cancer analysis of the oncogenic role of DLD across different tumors types. As the expression of DLD in ovarian cancer was high, and high expression is associated with poor prognosis, experimental verification of DLD in ovarian cancer was conducted. In the present study, DLD expression was found to be high in the ovarian cancer OC3 cell line, compared with the normal ovarian epithelial IOSE80 cell line by reverse transcription-quantitative PCR analysis. After knockdown of DLD expression, it was found that DLD regulated metabolic pathways by suppressing the intracellular NAD^+^/NADH ratio, which then in turn suppressed tumor cell proliferation detected by MTT assay. In conclusion, the present pan-cancer analysis of DLD demonstrated that DLD expression was associated with the clinical prognosis, immune infiltration and tumor mutational burden in 33 tumor types, and experimental verification in ovarian cancer was conducted. These results may contribute to the understanding of the role of DLD in tumorigenesis.

## Introduction

Copper is an essential micronutrient and is required for a wide range of physiological processes in all cell types in humans. Therefore, copper homeostasis is highly important and the accumulation of intracellular copper can lead to oxidative stress, resulting in perturbed cellular function. Recently, a new pattern of cell death, named copper-dependent death, has been reported and its mechanism was distinct from other mechanisms known to regulate cell death as it was demonstrated to occur through the direct binding of copper to lipid components of the tricarboxylic acid (TCA) cycle (1). The dihydrolipoamide dehydrogenase (DLD) gene encodes a member of the class-I pyridine nucleotide-disulfide oxidoreductase family, and is a key regulator of the TCA cycle that has also been identified as a moonlighting protein based on its ability to regulate energy metabolism and the cell cycle (2). In the TCA cycle, DLD catalyzes the reduction of NAD^+^ to NADH in the second dehydration step (3). Previous studies have found that DLD is one of seven genes (FDX1, LIPT1, LIAS, DLD, DLAT, PDHA1, and PDHB) that are involved in copper-dependent death (1,4). Among these genes, FDX1 is a reductase known to reduce Cu^2+^ to its more toxic form, Cu^+^, and to be a direct target of elesclomol (4). LIPT1, LIAS, and DLD are components of the lipoic acid pathway, and DLAT, PDHA1, and PDHB are protein targets of lipoylation (1,4,5). Tumor cells depend on glutaminolysis fuelling to carry out the TCA cycle and essential biosynthetic processes supporting tumor growth, and DLD, a dehydrogenase found in several multi-enzyme complexes that regulate energy metabolism, plays an important role in the tumor biological process (2). It is therefore important to perform a pan-cancer expression analysis of copper-induced cell death-associated genes. This information can be used to assess their association with clinical prognosis and asses their underlying molecular functions mechanistically. A pan-cancer analysis of FDX1 was recently conducted, and it was demonstrated that FDX1 was significantly associated with immune-related pathways (6). The expression level of FDX1 was also demonstrated to be correlated with immune cell infiltration, immune checkpoint genes, and immune regulatory genes (6).

To the best of our knowledge, there is currently no pan-cancer evidence based on clinical big data that demonstrated the relationship between DLD and various tumor types. Therefore, the aim of the present study was to use publicly available databases to conduct a pan-cancer analysis of DLD, including an analysis of the DLD expression profile, DLD genetic alteration associated with survival status and the degree of immune infiltration. In addition, the present study attempted to assess the possible underlying molecular mechanisms of DLD in different cancer types.

## Materials and methods

### Gene expression

In order to explore DLD expression in pan-cancer, DLD was first inputted into the ‘Gene DE’ module in tumor immune estimation resource, version 2 (TIMER2; http://timer.cistrome.org/), before the differential expression of DLD was observed between adjacent normal and tumor tissues, which compares data from The Cancer Genome Atlas (TCGA) project. The ‘Expression Analysis-Box Plots’ module in gene expression profiling interactive analysis, version 2 (GEPIA2: http://gepia2.cancer-pku.cn) was used to obtain the differential expression of DLD between the tumor tissues and the corresponding normal tissues. Using GEPIA, expression data in normal tissues were obtained from the genotype-tissue expression (GTEx) database and TCGA normal data, whereas tumor tissues data were obtained from the TCGA. In addition, violin plots of DLD expression at different pathological stages of the tumors were obtained from GEPIA2 in the ‘stage plots’ module, an interactive web resource for analyzing cancer Omics data. The University of Alabama at Birmingham CANcer data analysis Portal (UALCAN; http://ualcan.path.uab.edu/) was used for DLD protein expression analysis using the ‘CPTAC’ module.

### Survival prognosis

The ‘Survival Map’ module of GEPIA2 was used to obtain the overall survival (OS) and disease-free survival (DFS) significance of DLD in 33 tumor types. In the ‘Group Cutoff’ module ‘Median’ was selected, then high (≥50%) and low (<50%) cut-off values of the expression threshold were used to split the cohorts into high and low expression groups.

### Genetic alteration

In cBioPortal web (https://www.cbioportal.org/), ‘DLD’ was entered into the ‘Quickselect’ section before ‘TCGA Pan Cancer Atlas Studies’ was selected. The alteration frequency, mutation type and Copy Number alteration (CNA) of DLD across all TCGA tumors were found in the ‘Cancer Types Summary’ module. The ‘Comparison’ module was then used to obtain the data on the OS, DFS, progression-free, and disease-free survival differences, in addition to the Kaplan-Meier plots with log-rank P-values.

### Immune infiltration

In the TIMER2 web server, the ‘Immune-Gene’ module was used to explore the association between the degree of immune infiltration and DLD expression in tumors. Spearson's correlation analysis in TIMER2 was used to assess this association. As cancer-associated fibroblasts and CD8^+^ play a key role in the tumor microenvironment, cancer-associated fibroblasts and CD8^+^ T-cells were selected for analysis. The ‘TIMER’, ‘CIBERSORT-ABS’, ‘CIBERSORT’, ‘XCELL’, ‘QUANTISEQ’, ‘EPIC’, and ‘MCPCOUNTER’ algorithms were applied for the estimation of immune infiltration. A heatmap and a scatter plot were generated for the data.

### DLD-related gene enrichment analysis

A single protein name ‘DLD’ was inputted whereas the organism ‘Homo sapiens’ was selected in the Search Tool for the Retrieval of Interacting Genes/Proteins (STRING, version 11.5; http://string-db.org/) online tool. The main parameters were as follows: i) ‘Low confidence (0.150)’; ii) ‘evidence’; iii) ‘no more than 50 interactors’; and iv) ‘experiments’. Finally, a list of potential DLD-binding proteins were obtained. The top 100 DLD-correlated targeting genes were also obtained using the ‘Similar Gene Detection’ module of GEPIA, and a threshold of 0.6. In addition, in TIMER2, using the ‘gene_corr’ module, gene scatter plots and a heatmap of ‘purity’ and ‘infiltration level’ were obtained. Jvenn (7) was then used to compare the DLD-binding proteins from STRING and to obtain the related genes from GEPIA, and the overlap areas represented the common genes of the two groups. In addition, Kyoto Encyclopedia of Genes and Genomes (KEGG) (https://www.genome.jp/kegg/) was used for pathway analysis, using the threshold of a count >3, and P<0.05. The enriched pathways were visualised with the ‘ggplot2’ R packages (8).

### Cell culture

The human ovarian cancer cell line OC3 (cat. no. HTB-161) was obtained from American Type Culture Collection and the normal ovarian epithelial cell line IOSE80 (cat. no. CM-H049) was obtained from Shanghai Gaining Biotechnology Co., Ltd. Dulbecco's modified Eagle's medium (DMEM) (Invitrogen; Thermo Fisher Scientific, Inc.) supplemented with 10% Fetal Bovine Serum (FBS) (Invitrogen; Thermo Fisher Scientific, Inc.) and 1% penicillin/streptomycin were used to culture the cells at 37°C in a 5% CO_2_ incubator.

### siRNA transfection

Small interfering RNA (siRNA) was purchased from Shanghai GenePharma Co., Ltd. Transfections were performed as described previously (9). OC3 cells were cultured in DMEM supplemented with 10% FBS in a 6-well plate until 70% confluence, then transfected with siRNA (20 nM) using Lipofectamine^®^ 3000 (Invitrogen; Thermo Fisher Scientific, Inc.), according to the manufacturer's instructions. Transfection was performed at 37°C for 6 h before changing the transfection medium with fresh culture medium. The cells were harvested 48 h post-transfection for reverse transcription-quantitative PCR (RT-qPCR) analysis. The sequences used were listed in Table I.

### RT-qPCR

The total RNA was extracted from IOSE80 and OC3 cells using TRIzol^®^ reagent (Invitrogen; Thermo Fisher Scientific, Inc.), including both siRNA transfected and untransfected cells. After total RNA was extracted, the EasyScript One Step gDNA Removal and cDNA Synthesis SuperMix (TransGen Biotech Co., Ltd.) was used to acquire the first strand cDNA following the manufacturer's protocols. Next, qPCR was performed using TB Green^®^ Premix EX Taq™ II (Takara Bio, Inc.) with an Applied Biosystems 7900 Real-Time PCR System (Thermo Fisher Scientific, Inc.) with an initial denaturation at 95°C for 30 sec, followed by 95°C for 5 sec and 60°C for 34 sec by 40 cycles and extension at 72°C for 5 min; final extension at 72°C for 15 min. The internal RNA standard was β-actin expression. Expression levels of RNA were calculated based on the comparative 2^−ΔΔCq^ method (10). The primers used for RT-qPCR were provided in Table II. All reactions were performed in triplicate.

### Measuring the intracellular NAD^+^/NADH ratio

NAD^+^/NADH assay kit (cat. no. MET-5018; Cell Biolabs, Inc.) was used to measure the intracellular NAD^+^/NADH ratio following the literature reported protocol (11). For NAD measurement, siRNA transfected and untransfected OC3 cells grown in a 48-well plate were killed with 50 µl HClO_4_ and then neutralized with an equal volume of KOH, whereas the opposite was conducted for NADH extraction with an additional heating step (60°C, 30 min) between KOH and HClO_4_ additions. After the addition of 100 µl 100 mM bicine (pH 8), 50 µl cell extract was mixed with an equal volume of bicine buffer containing 23 µl/ml ethanol, 0.17 mg/ml MTT, 0.57 mg/ml phenazine ethosulfate, and 10 µg of alcohol dehydrogenase. Finally, a microplate reader was used to measure the absorption at 450 nm after 1 h incubation at room temperature.

### Cell proliferation assay

Post-transfection, OC3 cells were seeded at a density of 1,000 cells per well in 96-well plates. A total of five wells were used as replicates. A total of 10 µl MTT reagent (5 mg/ml) was incubated with the cells (0, 24, 48, and 72 h groups) at 37°C for 2 h. Subsequently, 150 µl DMSO was added to each of the wells. A microplate reader was used to measure the absorbance at a wavelength of 490 nm.

### Statistical analysis

GraphPad Prism 8.0 (GraphPad Software, Dotmatics, Inc.) was used to analyse the data using an unpaired Student's t-test. Data are presented as the mean ± standard deviation. P<0.05 was considered to indicate a statistically significant difference. All proliferation experiments were performed in triplicate.

## Results

### Gene expression analysis

**Figure 1.**
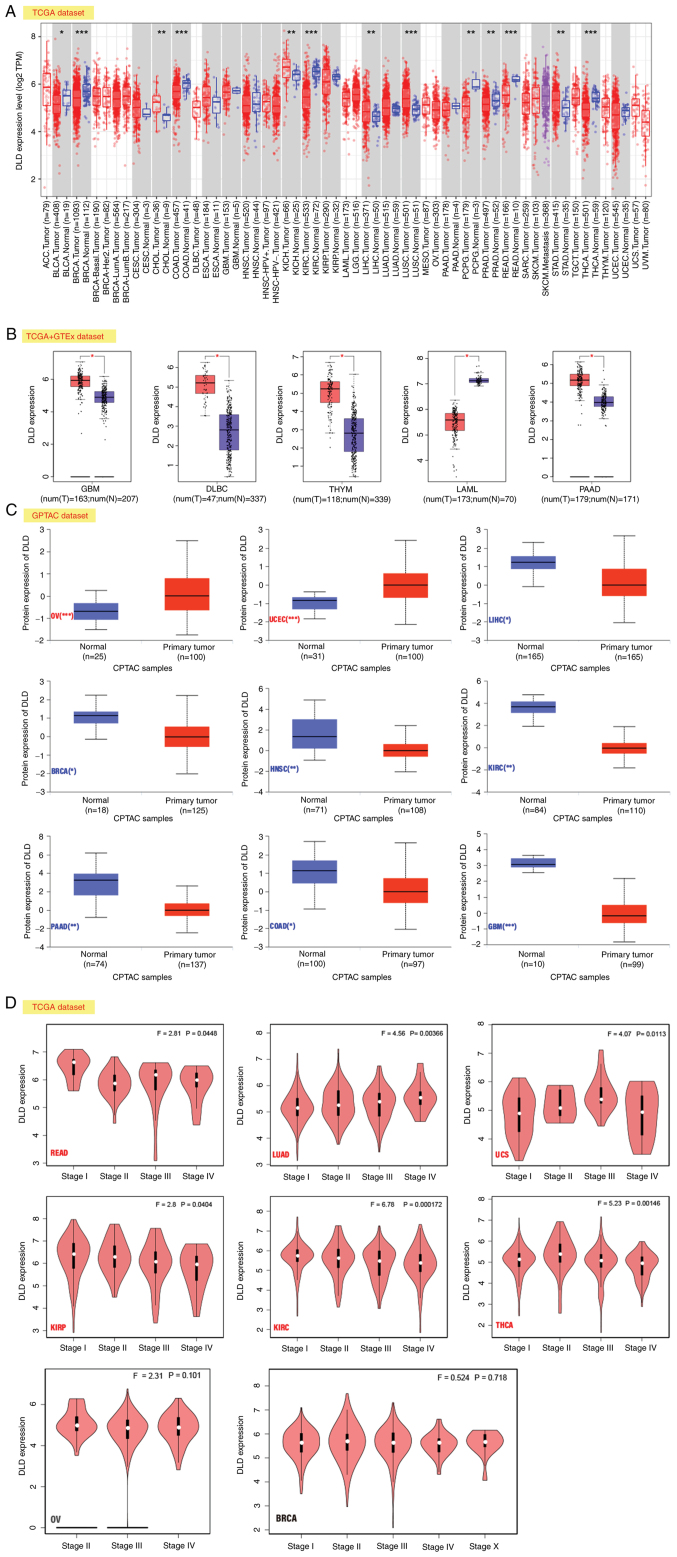
Expression level of DLD. (A) DLD expression in different cancers according to TIMER2. (B) DLD expression levels in GBM, DLBC, THYM, LAML, and PAAD. Data were taken from TCGA and GTEx databases. The red color indicates DLD high expression, the blue color indicates DLD low expression. (C) Comparison of total DLD protein expression levels between normal tissue and primary tumor tissue according to the CPTAC dataset for OV, UCEC, LIHC, BRCA, HNSC, KIRC, PAAD, COAD, GBM. (D) Expression levels of the DLD at different pathological stages in READ, LUAD, UCS, KIRP, KIRC, THCA, OV and BRCA based on TCGA data. *P<0.05; **P<0.01; ***P<0.001. DLD, Dihydrolipoamide dehydrogenase; TIMER2, tumor immune estimation resource; TCGA, The Cancer Genome Atlas; GTEx, genotype-tissue expression; GBM, glioblastoma Multiforme; DLBC, lymphoid neoplasm diffuse large B-cell lymphoma; THYM, thymoma; UCEC, uterine corpus endometrial carcinoma; LAML, acute myeloid leukemia; READ, rectum adenocarcinoma; PAAD, pancreatic adenocarcinoma; LUAD, lung adenocarcinoma; UCS, uterine carcinosarcoma; KIRP, kidney renal papillary cell carcinoma; LIHC, liver hepatocellular carcinoma; BRCA, breast invasive carcinoma; HNSC, head and neck squamous cell carcinoma; KIRC, kidney renal clear cell carcinoma; THCA, thyroid carcinoma; COAD, colon adenocarcinoma; OV, ovarian serous cystadenocarcinoma.

In the TCGA datasets, it was found that in Cholangiocarcinoma (CHOL), liver hepatocellular carcinoma (LIHC), lung squamous cell carcinoma (LUSC), kidney chromophobe (KICH) and stomach adenocarcinoma (STAD), the expression of DLD was higher compared with that in the corresponding control tissues. However, in bladder urothelial carcinoma (BLCA), colon adenocarcinoma (COAD), breast invasive carcinoma (BRCA), kidney renal clear cell carcinoma (KIRC), pheochromocytoma and paraganglioma (PCPG), rectum adenocarcinoma (READ), prostate adenocarcinoma (PRAD), and thyroid carcinoma (THCA), the expression of DLD was lower compared with that the corresponding control tissues (Fig. 1A).

The expression profiles of DLD were then evaluated after including the dataset of normal tissues from GTEx, which found that DLD expression was higher in glioblastoma multiforme (GBM), pancreatic adenocarcinoma (PAAD), thymoma (THYM), and lymphoid neoplasm diffuse large B-cell lymphoma (DLBC). However, the opposite was found in acute myeloid leukemia (LAML; Fig. 1B).

In ovarian serous cystadenocarcinoma (OV) and uterine corpus endometrial carcinoma (UCEC), the expression of DLD was higher compared with that in the normal tissues according to the results of the CPTAC dataset. By contrast, the expression of DLD was lower in LIHC, BRCA, head and neck squamous cell carcinoma (HNSC), PAAD, KIRC, COAD and GBM compared with that in normal tissues (Fig. 1C).

In READ, lung adenocarcinoma (LUAD), kidney renal papillary cell carcinoma (KIRP), uterine carcinosarcoma (USC), and thyroid carcinoma (THCA), the association between DLD expression and the pathological stages was found to be statistically significant according to the analysis by the ‘Pathological Stage Plot’ module of GEPIA. However, in OV and BRCA, there were no significant associations (Fig. 1D).

The analysis in Fig. 1A is from mRNA expression data obtained from the TCGA database but the analysis in Fig. 1C is from protein expression data obtained from the CPTAC database. However, Fig. 1D is an analysis of the association between DLD expression and pathological stage, so the expression levels of DLD in Fig. 1A, C and D were not similar.

### Survival analysis

**Figure 2.**
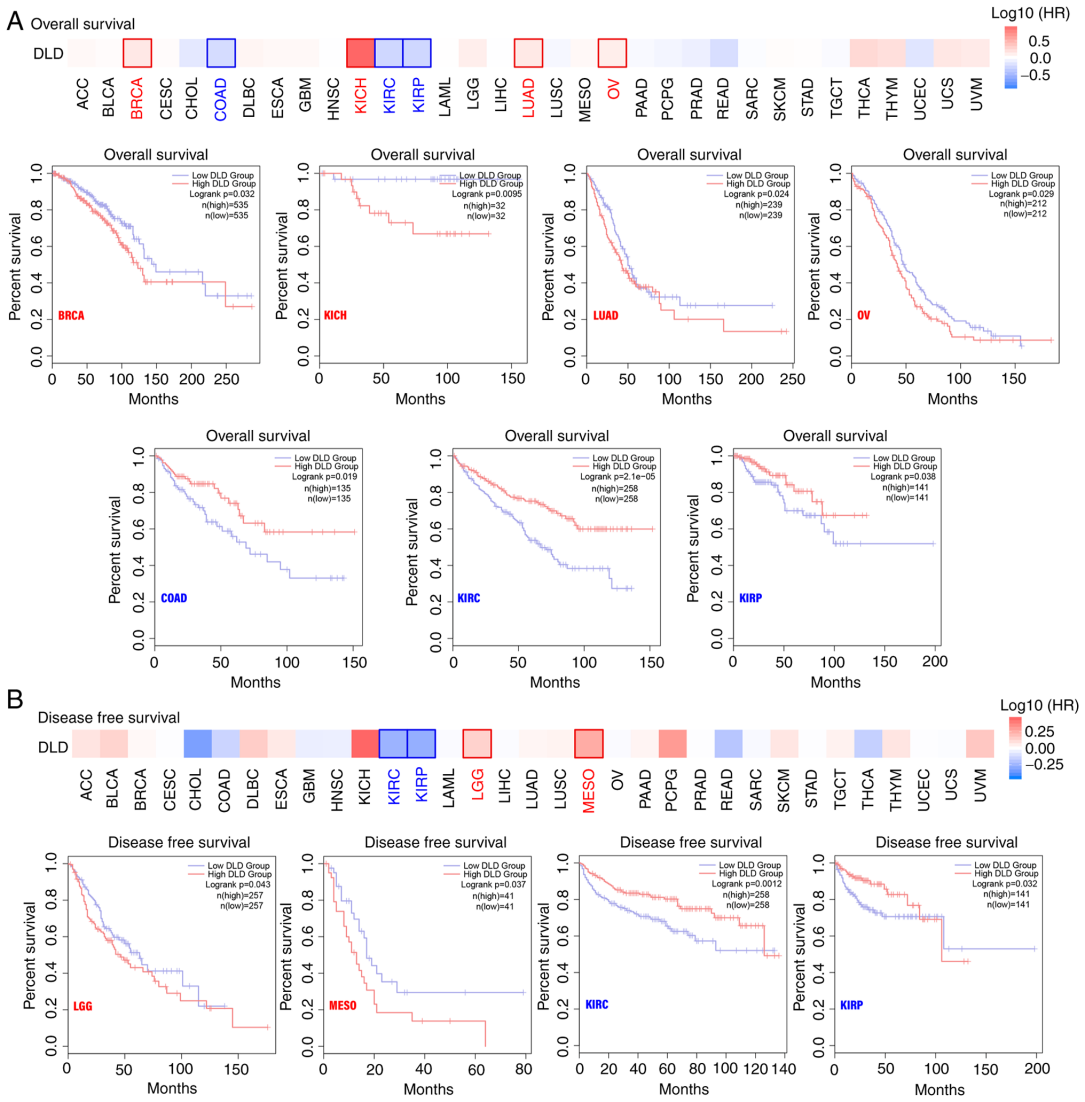
Relationship between DLD expression and patient prognosis. The GEPIA2 tool was used to perform (A) overall survival and (B) disease-free survival analysis. GEPIA, gene expression profiling interactive analysis; DLD, Dihydrolipoamide dehydrogenase; BRCA, breast invasive carcinoma; KICH, kidney chromophobe; LUAD, lung adenocarcinoma; OV, ovarian serous cystadenocarcinoma; COAD, colon adenocarcinoma; KIRC, kidney renal clear cell carcinoma; KIRP, kidney renal papillary cell carcinoma; LGG, brain lower grade glioma; MESO, mesothelioma.

TCGA datasets were divided into high and low expression groups according to DLD expression. It was found that in BRCA, KICH, LUAD and OV, highly expressed DLD was associated with poor prognosis, specifically OS. By contrast, lower DLD expression was associated with shorter OS in COAD, KIRC and KIRP (Fig. 2A). In terms of DFS, in lower grade glioma (LGG) and mesothelioma (MESO), higher DLD expression was associated with poorer prognosis. However, in KIRC and KIRP, lower expression of DLD was associated with poor prognosis (Fig. 2B). However, in Fig. 2 there was late time crossover of the curves, these events may affect the results of the log-rank analysis. A weighted test, such as the Renyi or Cramer-von Mises method, should be more appropriate (12). Since the figures were obtained from GEPIA, a new statistical analysis was not possible.

### Genetic alteration analysis

**Figure 3.**
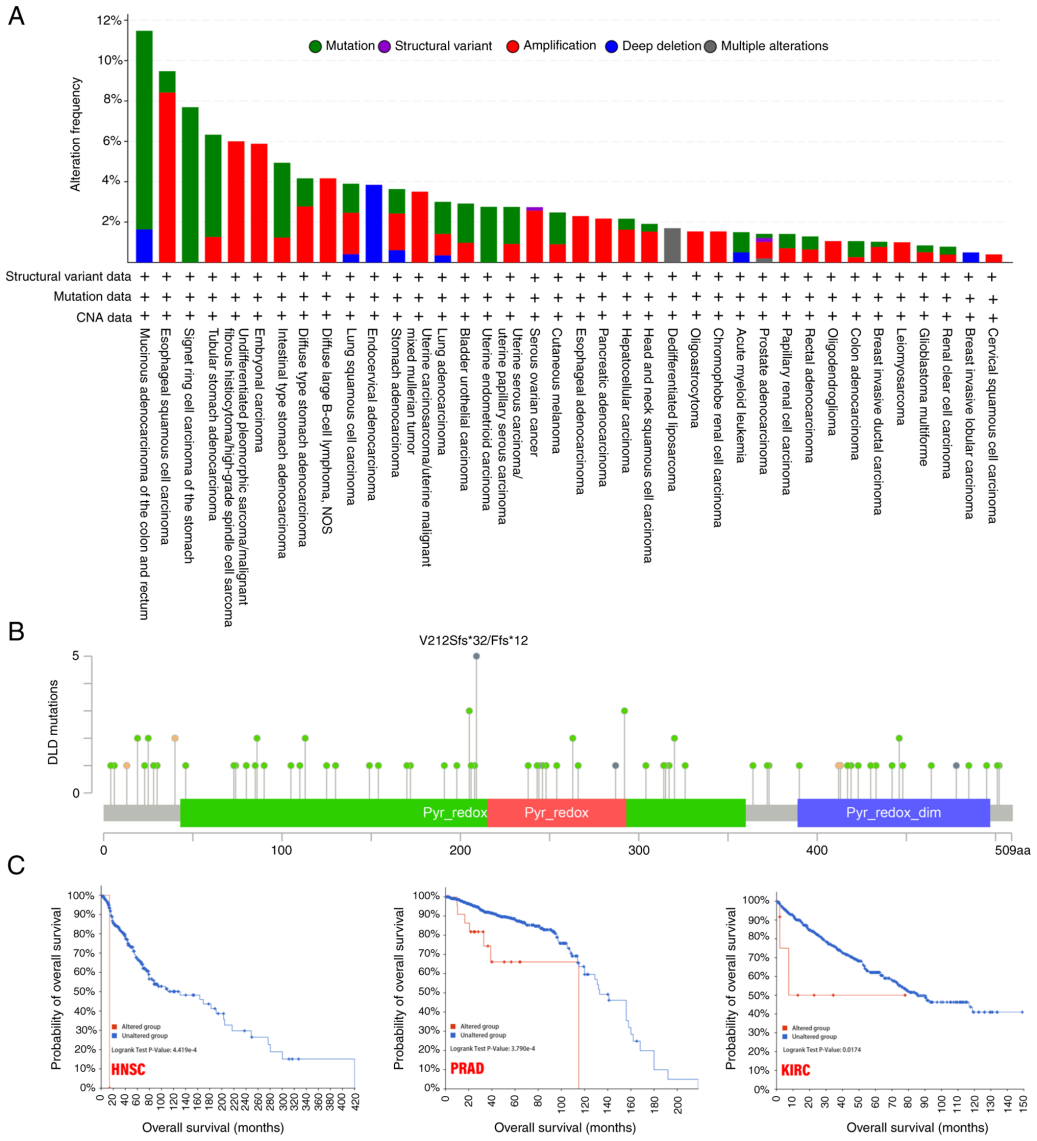
Mutation features of DLD. (A) The alteration frequency for each mutation type and (B) mutation site. (C) Potential association between mutation status and overall survival from HNSC, PRAD and KIRC, according to cBioPortal tool. DLD, Dihydrolipoamide dehydrogenase; HNSC, head and neck squamous cell carcinoma; PRAD, prostate adenocarcinoma; KIRC, kidney renal clear cell carcinoma.

In the different tumor samples, the frequency of genetic alterations in the DLD gene in a list of cancers was analyzed. The highest alteration frequency of DLD (>10%) appeared for patients with mucinous adenocarcinoma of the colon and rectum, with ‘mutation’ as the primary type. In the undifferentiated pleomorphic sarcoma/malignant fibrous histiocytoma/high-grade spindle cell sarcoma cases, ‘amplification’ was the primary type of alteration, with a frequency of ~6%. In endocervical adenocarcinoma cases, the copy number deletion of DLD was found to be the most dominant genetic alteration, with a ~4% frequency (Fig. 3A).

The V212Sfs*32/Ffs*12 alteration in the Pyr_redox domain was found to be the main type of genetic alteration of DLD (Fig. 3B). After exploring the clinical survival prognosis of tumors associated with genetic alterations of DLD, the results showed that in HNSC, PRAD and KIRC, the genetic alterations group were associated with poorer prognosis in terms of OS (Fig. 3C).

### Immune infiltration analysis

**Figure 4.**
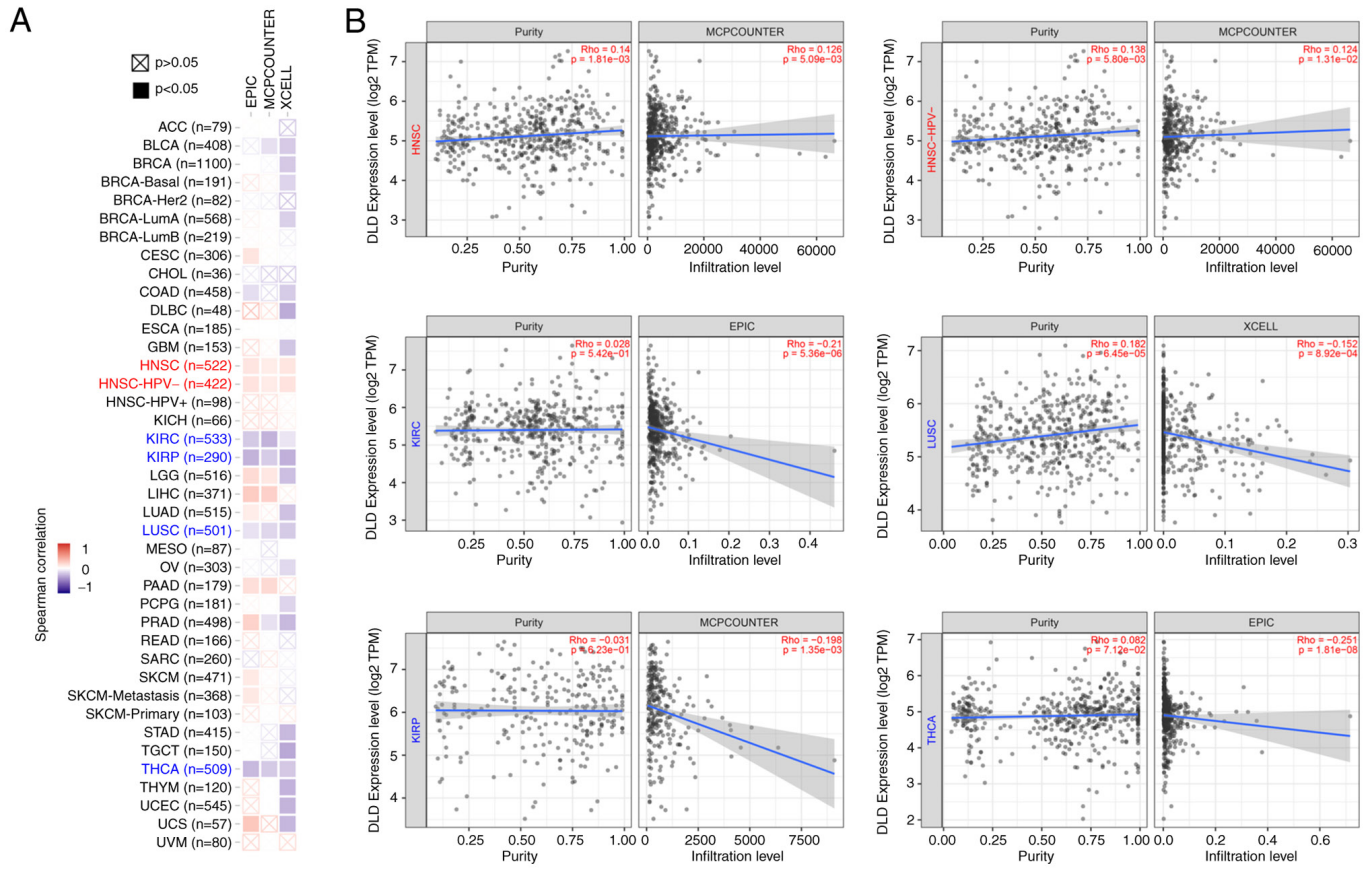
Relationship between DLD expression and immune infiltration of fibroblasts associated with cancer. (A) Heatmap showing the correlation between DLD expression and infiltration levels of cancer-associated fibroblasts data. (B) Correlation between DLD expression and the degree of infiltration by cancer-associated fibroblasts in HNSC, HNSC- HPV-, KIRC, LUSC, KIRP and THCA. DLD, Dihydrolipoamide dehydrogenase; HNSC, head and neck squamous cell carcinoma; HNSC-HPV, head and neck squamous cell carcinoma with human papillomavirus; KIRC, kidney renal clear cell carcinoma; LUSC, lung squamous cell carcinoma; KIRP, kidney renal papillary cell carcinoma; THCA, thyroid carcinoma.

Tumor-infiltrating immune cells have been previously implicated in tumor initiation, progression and metastasis (13). Cancer-associated fibroblasts can also infiltrate tumors (14). A positive correlation between DLD expression and the estimated degree of infiltration by cancer-associated fibroblasts was found in HNSC, and HNSC without human papillomavirus (HPV) infection. However, there was a negative correlation between DLD expression and cancer-associated fibroblast infiltration in KIRC, LUSC, KIRP and THCA (Fig. 4A). Corresponding scatterplot data of the aforementioned cancers for the correlation between DLD expression and the estimated infiltration value were also represented (Fig. 4B).

### Enrichment analysis

**Figure 5.**
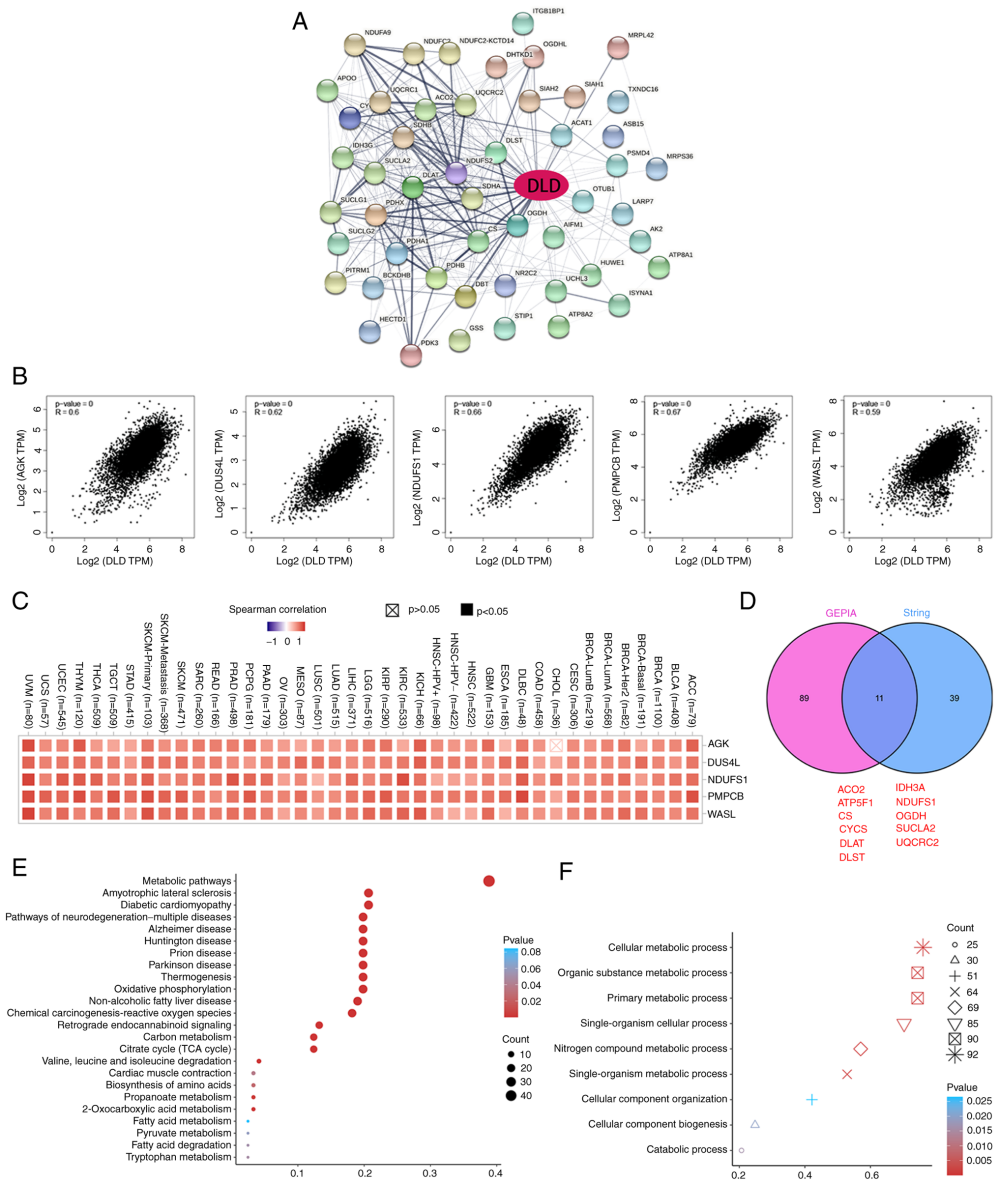
DLD-related gene enrichment analysis. (A) Potential DLD-binding proteins were predicted using the Search Tool for the Retrieval of Interacting Genes/Proteins tool. Thickness of edge indicates the strength of the correlation. (B) Top five DLD-correlated genes were found using the gene expression profiling interactive analysis project. Correlation between DLD expression and that of the selected target genes AGK, DUS4L, NDUFS1, PMPCB and WASL are shown. (C) Corresponding heatmap data for AGK, DUS4L, NDUFS1, PMPCB and WASL in a list of cancers. (D) DLD-binding proteins and DLD-correlating genes were overlapped by intersection analysis. (E) Kyoto Encyclopedia of Genes and Genomes pathway analysis. (F) Molecular function data in Gene Ontology analysis. DLD, Dihydrolipoamide dehydrogenase; AGK, acylglycerol kinase; DUS4L, dihydrouridine synthase 4-like; NDUFS1, NADH dehydrogenase Fe-S protein 1; PMPCB, peptidase mitochondrial processing β; WASL, Wiskott-Aldrich syndrome-like. ACO2, aconitase 2; ATP5F1, mitochondrial Fo complex subunit B1; CS, citrate synthase; CYCS, cytochrome c; DLAT, dihydrolipoamide S-acetyltransferase; DLAST, dihydrolipoamide S-succinyltransferase; IDH3A, isocitrate dehydrogenase 3α; OGDH, oxoglutarate dehydrogenase; SUCLA2, succinate-CoA ligase; UQCRC2, ubiquinol-cytochrome c reductase core protein II.

A total of 50 potential DLD-binding proteins were obtained from the STRING tool to investigate the molecular mechanisms of DLD in tumorigenesis (Fig. 5A). The top 100 genes associated with DLD expression were also obtained based on GEPIA. acylglycerol kinase (AGK), dihydrouridine synthase 4-like (DUS4L), NADH dehydrogenase Fe-S protein 1 (NDUFS1), peptidase mitochondrial processing β (PMPCB), and Wiskott-Aldrich syndrome-like (WASL) were the top five genes that positively associated with DLD expression (Fig. 5B). The corresponding heatmap data also showed a positive correlation between DLD expression and that of the aforementioned five genes in the majority of the cancer types tested (Fig. 5C). aconitase 2, mitochondrial Fo complex subunit B1, citrate synthase, cytochrome c, DLAT, dihydrolipoamide S-succinyltransferase, isocitrate dehydrogenase 3α, NDUFS1, oxoglutarate dehydrogenase, succinate-CoA ligase, and ubiquinol-cytochrome c reductase core protein II were found to be in common between the two STRING and GEPIA groups according to Venn analysis, which mean that these 11 genes were closely related to DLD (Fig. 5D). KEGG and gene ontology (GO) enrichment analyses were also performed. It was found that ‘metabolic pathways’ was the most enriched in the effect of DLD on tumor pathogenesis by KEGG (Fig. 5E). In addition, ‘cellular metabolic process’ was the pathway enriched in most genes by GO enrichment analysis (Fig. 5F).

### Knocking down DLD expression increases the NAD^+^/NADH ratio in OV

**Figure 6.**
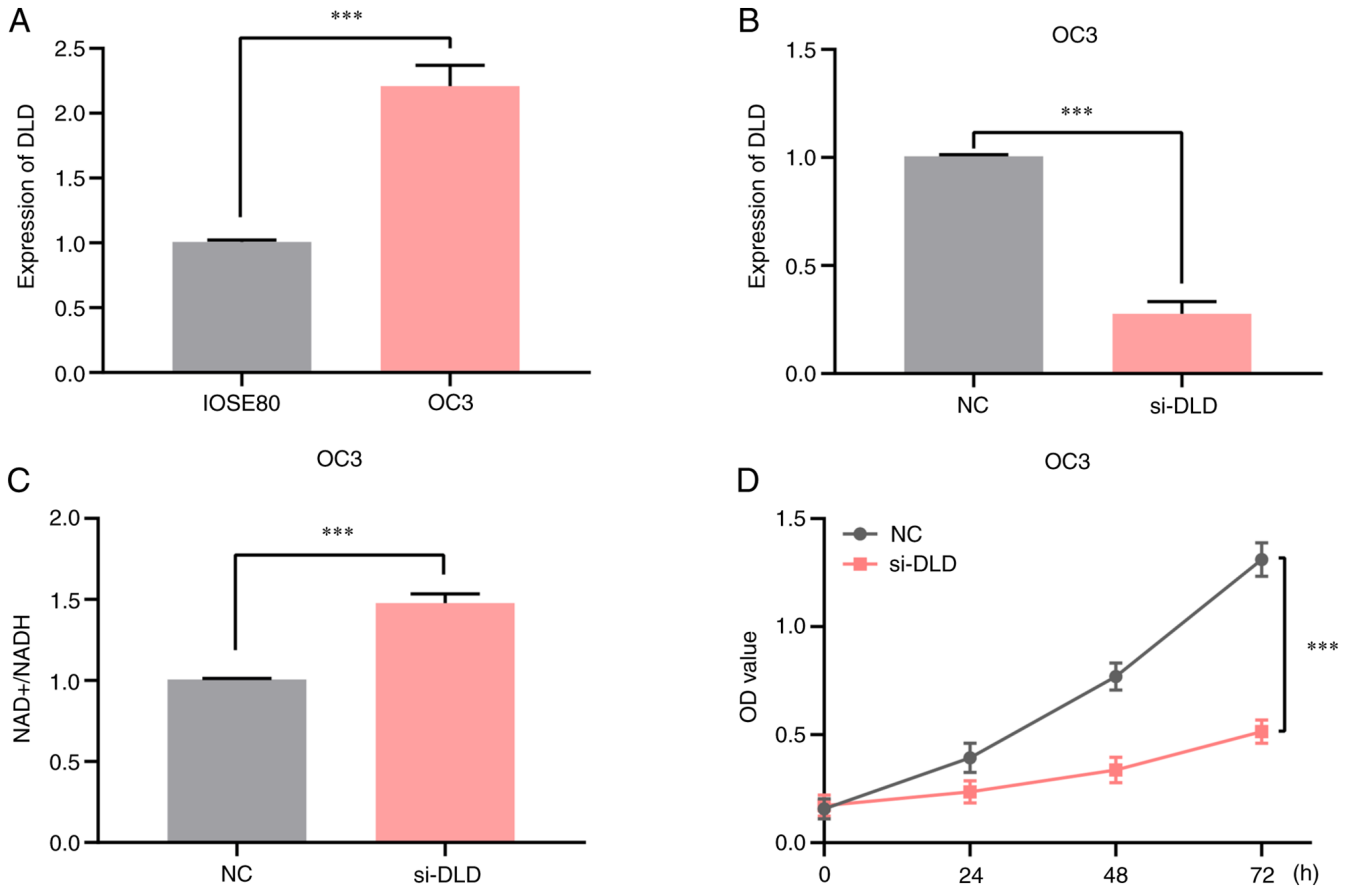
Knocking down DLD expression increases the NAD+/NADH ratio in OV. (A) The OV cell line OC3 exhibits higher DLD expression compared with that in the ovarian epithelial cell line IOSE80. (B) Transfection efficiency of si-DLD in the OC3 cell line by reverse transcription-quantitative PCR. (C) Effect of si-DLD knockdown on the intracellular NAD+/NADH ratio. (D) MTT assay of the effect of DLD-knockdown on cell proliferation in the OC3 cell line. ***P<0.001. DLD, Dihydrolipoamide dehydrogenase; OV, ovarian cancer; siRNA, small-interfering RNA. OD, optical density.

Because DLD was highly expressed in ovarian cancer and associated with a poor prognosis, OV cells were selected for *in vitro* experiments. It was showed by RT-qPCR analysis that the mRNA expression levels of DLD in OC3 cells were significantly higher compared with those in the normal ovarian epithelial cell line IOSE80 (Fig. 6A). Next, the efficiency of the DLD-knockdown was assessed by RT-qPCR (Fig. 6B), and it was found that DLD expression was successfully decreased. In addition, a significant increase in the NAD^+^/NADH ratio was observed in OC3 cell line compared with that in the negative control group, when DLD expression was knocked down by DLD-specific siRNA (Fig. 6C), suggesting that the intracellular NAD^+^/NADH ratio was regulated by DLD inhibition. Results of the MTT assay indicated that DLD-knockdown inhibited OC3 cell proliferation (Fig. 6D). Thus, these findings suggest that suppressing DLD expression can increase the NAD^+^/NADH ratio in OV cells, which inhibited OC3 cell proliferation.

## Discussion

A novel form of cell death induced by intracellular copper has been demonstrated in a recent study, which has been termed ‘cuproptosis’ and has been previously shown to occur through affecting TCA cycle (1). Cuproptosis operates through the binding of copper to one of the lipoylated components (in particular, the pyruvate dehydrogenase complex) of the TCA cycle, and DLD is a key regulator of the TCA cycle (15). DLD has been reported to participate in a variety of cellular processes in humans, such as regulating energy metabolism and the cell cycle (16–18). To the best of our knowledge, the functional relationship between DLD and tumors has only been reported in four publications to date (19–22). Therefore, it remains to be fully elucidated whether DLD may serve significant functions in tumors. Through a literature search based on the pan-cancer perspective, no evidence of a pan-cancer analysis of DLD was found. Therefore, in a total of 33 different tumors, the DLD gene expression profile was comprehensively examined in the present study based on the online Bioinformatics databases, where gene expression, genetic alteration, and immune infiltration were among the parameters included.

In the present study, DLD was found to be highly expressed in a number of tumors. Higher levels of DLD expression in tumors from CHOL, LIHC, LUSC, KICH, and STAD were found compared with those in the corresponding normal tissues according to analysis in TIMER2, using data from TCGA. However, the findings using the CPTAC dataset revealed that in OV and UCEC the expression of DLD was higher compared with that in normal tissues. Since the TCGA database was constructed based on the RNA expression level whereas the data in GPTAC database were based on protein expression level, the results were not consistent. This finding suggested that DLD expression is likely to be part of a complex functional network in tumors, on both RNA and protein levels. Nevertheless, for survival prognosis, GEPIA was used in the present study to assess the association between DLD expression and survival. In patients with BRCA, LUAD, KICH and OV, higher expression of DLD was associated with poorer prognosis for OS. For DFS, higher DLD expression was associated with poorer prognosis in patients with LGG and MESO. This inconsistency may be attributed to the different carcinogenic mechanisms in the different tumors, which require further study.

Tumor mutations have been noted for >50 years. Mutations have been reported to serve an important role in the occurrence and development of tumors, which have been proposed to be an important target for tumor diagnosis and treatment (23). Through the present analysis, it was found that DLD mutations were also abundant in the different tumors. In particular, patients with mucinous adenocarcinoma of the colon and rectum had the highest frequency of alterations in DLD with the ‘mutation’ type, whereas the ‘amplification’ type of mutation was frequent in the undifferentiated pleomorphic sarcoma/malignant fibrous histiocytoma/high-grade spindle cell sarcoma case. In HNSC, PRAD and KIRC, high mutation rates were also associated with poorer prognosis.

To the best of our knowledge, the present study also found for the first time evidence of a correlation between the extent of immune infiltration and DLD expression across some tumor types. Immune infiltration and the tumor microenvironment have been shown to serve a function in the development of various tumors by regulating the dynamic interaction among each of their molecular features. Specifically, the immune microenvironment has a large influence on the clinical outcomes (24). Cancer-associated fibroblasts have been reported to be present in the stroma of the tumor microenvironment, where they can modulate the function of numerous tumor-infiltrating immune cells (14). It was also found that DLD can exert an important role by regulating immune infiltration and the tumor microenvironment in the present study through using the TIMER database. After a series of analyses in the TIMER database, it was found that there was a positive correlation between DLD expression and the estimated infiltration levels by cancer-associated fibroblasts in HNSC and HNSC-HPV-negative. However, a negative correlation between these two parameters was noted in KIRC, LUSC, KIRP, and THCA. In addition, the present findings suggested for the first time, to the best of our knowledge, that DLD expression was associated with the level of infiltration by cancer-associated fibroblasts in HNSC, KIRC, LUSC, KIRP, and THCA.

Subsequently, information on the DLD expression-related genes were integrated across all tumors for a range of enrichment analyses, which identified the potential effects of ‘metabolic pathways’ and ‘cellular metabolic process’ in its role in the etiology or pathogenesis of cancer. These results were similar to a recent study, which reported that DLD can function as a novel metabolic pathway protein regulating the TCA in human melanoma progression (20).

Based on the bioinformatics analysis in the present study, it was found that DLD may regulate ‘metabolic pathways’ and ‘cellular metabolic process’ in cancer. The bioinformatics analysis results also showed a high level of DLD expression in OV, with DLD expression being associated with poor prognosis. In addition, higher expression of DLD in the OV cell line was found compared with that in the normal ovarian cell line. When DLD expression was knocked down by siRNA in the OC3 cell line, a significant increase in the NAD^+^/NADH ratio was found compared with that in the negative control group. Several studies have reported that the NAD^+^/NADH ratio may affect tumor progression (25,26). Therefore, these results suggest that DLD can regulate metabolic pathways in OV by suppressing the intracellular NAD^+^/NADH ratio.

In summary, the present pan-cancer analysis of DLD showed that DLD expression was associated with the clinical prognosis, immune infiltration, and tumor mutational burden in some tumor types. This may contribute to understanding the role of DLD in tumorigenesis from the perspective of clinical tumor samples.

## Figures and Tables

**Table I.** siRNA and negative control sequences.

Name	Sequence (5′-3′)
DLD siRNA	Sense: GCACUAAUGUGUAAGACAA
	Antisense: UUGUCUUACACAUUAGUGC
Control DLD siRNA	AUGAUGGCACGUCGUACAC

DLD, Dihydrolipoamide dehydrogenase; siRNA, small interfering RNA.

**Table II.** Primers for reverse transcription-quantitative PCR.

Gene	Primer sequence (5′-3′)
Dihydrolipoamide	F: TTACACACACCCTGAAGTTGC
dehydrogenase	R: GGATCTTCACCATGCCATCTG
β-actin	F: TCACCCACACTGTGCCCATCTACGA
	R: CAGCGGAACCGCTCATTGCCAATGG

## Data Availability

The datasets used and/or analyzed during the current study are available from the corresponding author on reasonable request.
